# Overexpression of vascular endothelial growth factor C increases growth and alters the metastatic pattern of orthotopic PC-3 prostate tumors

**DOI:** 10.1186/1471-2407-9-362

**Published:** 2009-10-12

**Authors:** Johanna Tuomela, Maija Valta, Jani Seppänen, Kati Tarkkonen, H Kalervo Väänänen, Pirkko Härkönen

**Affiliations:** 1 Institute of Biomedicine, Department of Cell Biology and Anatomy, University of Turku, Finland; 2 Orthotopix Ltd, Turku Finland; 3 Department of Laboratory Medicine, Tumor Biology, MAS University Hospital, Lund University, Sweden

## Abstract

**Background:**

Prostate cancer metastasizes to regional lymph nodes and distant sites but the roles of lymphatic and hematogenous pathways in metastasis are not fully understood.

**Methods:**

We studied the roles of VEGF-C and VEGFR3 in prostate cancer metastasis by blocking VEGFR3 using intravenous adenovirus-delivered VEGFR3-Ig fusion protein (VEGFR3-Ig) and by ectopic expression of VEGF-C in PC-3 prostate tumors in nude mice.

**Results:**

VEGFR3-Ig decreased the density of lymphatic capillaries in orthotopic PC-3 tumors (*p *< 0.05) and inhibited metastasis to iliac and sacral lymph nodes. In addition, tumor volumes were smaller in the VEGFR3-Ig-treated group compared with the control group (*p *< 0.05). Transfection of PC-3 cells with the VEGF-C gene led to a high level of 29/31 kD VEGF-C expression in PC-3 cells. The size of orthotopic and subcutaneous PC-3/VEGF-C tumors was significantly greater than that of PC-3/mock tumors (both *p *< 0.001). Interestingly, while most orthotopic PC-3 and PC-3/mock tumors grown for 4 weeks metastasized to prostate-draining lymph nodes, orthotopic PC-3/VEGF-C tumors primarily metastasized to the lungs. PC-3/VEGF-C tumors showed highly angiogenic morphology with an increased density of blood capillaries compared with PC-3/mock tumors (*p *< 0.001).

**Conclusion:**

The data suggest that even though VEGF-C/VEGFR3 pathway is primarily required for lymphangiogenesis and lymphatic metastasis, an increased level of VEGF-C can also stimulate angiogenesis, which is associated with growth of orthotopic prostate tumors and a switch from a primary pattern of lymph node metastasis to an increased proportion of metastases at distant sites.

## Background

A high proportion of prostate cancer patients have metastatic disease, which causes high morbidity and mortality. Tumor spread takes place primarily via lymphatic vessels, which leads to early metastases in prostate-draining iliac and sacral lymph nodes [1]. In advanced prostate cancer, tumors commonly metastasize to bone and other distant sites presumably via angiogenic routes [2,3]. The mechanisms causing invasive and metastatic prostate cancer are still largely unknown. The interactions of lymphangiogenic and angiogenic pathways in the development of distant metastases of prostate cancer are not fully understood either. Metastasis in regional lymph nodes is associated with later appearance of bone metastases and a poor prognosis of prostate cancer [4], but it is not known whether spreading to distant sites occurs via regional lymph nodes or directly via hematogenous routes [5].

Vascular endothelial growth factor C (VEGF-C) and its receptors VEGFR3 and VEGFR2 have a central role in regulation of the development and function of the lymphatic system as well as in tumor lymphangiogenesis. VEGF-C induces lymphatic endothelial cell proliferation, survival and migration *in vitro *[6,7]. It is a dimeric glycoprotein, which binds and activates VEGF receptor tyrosine kinases [8]. VEGF-C was first cloned from human prostate cancer cell line (PC-3 cells) as a ligand for lymphangiogenic receptor (VEGFR3) and angiogenic receptor (VEGFR2) [9]. It is synthesized intracellularly as a 58 kD propeptide, which undergoes proteolytic cleavage, and leads to 29 and 31 kD polypeptides, which are rapidly secreted and bind VEGFR3 in particular with high affinity [6]. Additional processing results in 21 kD VEGF-C, which binds both VEGFR2 and VEGFR3 [6].

Peritumoral lymphangiogenesis enables tumor cell dissemination to lymphatic vessels. VEGF-C is overexpressed in various human cancers including breast cancer [10-12] and prostate cancer [13-16]. In experimental models, tumor cells that overexpress VEGF-C induce peritumoral lymphangiogenesis and tumor cell invasion to lymphatic vessels [17-21]. However, conflicting data of this has been reported in other studies suggesting that intratumoral lymphatic vessels may not be functional or that peripheral lymphatics may not function properly, which questions the role of lymphangiogenesis in primary tumor cell dissemination [22,23]. The lymphangiogenic effects of VEGF-C can be blocked by VEGFR3 inactivation [24-26]. Without VEGF-C or VEGFR3, lymphatic endothelial cells are not able to form sprouting capillaries. In VEGF-C -/- mice, the sprouting of lymphatic vessels can be rescued by adding VEGF-C or VEGF-D (another lymphangiogenic growth factor that binds VEGFR3) but not VEGF, which indicates that the lymphangiogenic effects of VEGF-C are mediated primarily through VEGFR3 [27]. Like VEGF-C, VEGFR3 is upregulated in vascular endothelial cells in pathological conditions such as solid tumors [25,28-31].

Regulation of VEGF-C and VEGFR3 in prostate cancer metastasis was studied using orthotopic PC-3 prostate tumors, which naturally express VEGF-C. Orthotopic PC-3 tumors invade spontaneously to prostate-draining lymph nodes, but dissemination to distant sites such as bone, lung and liver occurs more slowly and less frequently [32]. We studied the effect of blockade of VEGFR3 on PC-3 tumor growth and metastasis by using adenoviral delivery of a VEGFR3 Ig fusion protein (VEGFR3-Ig), which functions as a decoy receptor for VEGFR3. Furthermore, we studied the effect of a high level of VEGF-C on growth, angiogenesis, lymphangiogenesis and metastasis in orthotopic PC-3 prostate tumors, expressing ectopic VEGF-C.

## Methods

### Cell culture and transfection

The human hormone-resistant prostate cancer cell line PC-3 was obtained from the American Tissue Type Culture Collection (Rockville, MD, USA) and cultured in DMEM supplemented (10%) with heat inactivated fetal bovine serum, iFBS. At near confluence, cells were harvested in trypsin/EDTA solution (Biochrom AG, Germany), washed with culture medium and finally suspended at a concentration of 1 × 10^6^/100 μL in sterile PBS for subcutaneous inoculation. For orthotopic inoculation, cells were suspended at a concentration of 5 × 10^5^/20 μL in a sterile dye solution consisting of PBS (Biochrom AG, Germany) with green food color 33022 (5 μg/mL; Roberts Oy, Finland) [32,33]. The cells were kept on ice until used for inoculation.

The expression vector pcDNA3.1(+) (Invitrogen, CA, USA) containing human full-length VEGF-C cDNA [9] in an ECORI site and an empty pcDNA3.1(+) vector (mock) were used. Plasmids (2 μg/well on a six-well plate) were transfected into PC-3 cells using Lipofectamine (Invitrogen, CA, U.S.A) according to the manufacturer's instructions. Twenty-four hours after transfection, the cells were transferred to selection medium containing neomycin (G418 500 μg/mL) for three weeks. Isolated colonies were cloned. Cell clones were characterized by Northern and Western blotting. Several VEGF-C positive clones were obtained and one VEGF-C overexpressing clone (clone 1, later called PC-3/VEGF-C) and one control clone (PC-3/mock) where selected for *in vivo *studies.

### RNA isolation and Northern blot analysis

Total RNA was extracted from PC-3/VEGF-C and PC-3/mock cells using the guanidinium isothiocyanate method [34]. Subsequently, 20 μg of RNA was separated by electrophoresis, stained with ethidium bromide and blotted on GeneScreen Plus nylon membrane (NEN Research Products, Boston, MA), using standard conditions suggested by the manufacturer. A cDNA insert [9] of human VEGF-C was [^32^P]-dCTP-labeled by the random priming method (Ready-to-go DNA labeling Beads, Amersham Pharmacia Biotech, Piscataway, NJ). Hybridization and exposure to X-ray film were carried out using conditions suggested by the manufacturer.

### Western blot analysis and enzyme-linked immunosorbent assay (ELISA) analysis

Serum-free DMEM conditioned by PC-3/VEGF-C and PC-3/mock cells was harvested from the cultures. Heparin-binding proteins were isolated from the supernatant with 100 μL of heparin-sepharose (1:1 slurry; Amersham Pharmacia Biotech, Piscataway, NJ, USA) overnight at 4°C. Heparin-sepharose beads were sedimented by centrifugation and washed three times with 20 mM Tris-HCl, pH 7.4, 300 mM NaCl. Heparin-sepharose-bound proteins conditioned by 3 × 10^6 ^cells were extracted by 5 min incubation in Laemmli sample buffer at 95°C and separated by gel electrophoresis (SDS-PAGE). After collecting the conditioned media, also the cells were lysed to Laemmli buffer and separated on gel similarly. After transfer to nitrocellulose membrane (Bio-Rad), VEGF-C (from conditioned media) or β-actin (from cell lysates) was detected using a 1:500 dilution of a rabbit polyclonal anti-human VEGF-C antibody (Abcam, UK) or 1:2000 mouse monoclonal anti-β-actin antibody (Sigma, St Louis, MO). Horseradish peroxidase-labeled anti-rabbit or anti mouse-IgG (Jackson Immunoresearch Laboratories Inc, USA) was used as a secondary antibody at 1:5000 dilutions. Protein bands were visualized using the ECL chemiluminescence detection system (Amersham Corp., Sweden) [19]. VEGF-C protein concentrations from cell lysates and 24 h conditioned media of cell clones were further quantified by ELISA (Bender MedSystems, Vienna, Austria) according to the manufacturer's instructions. Data was expressed as VEGF-C protein concentration (ng/mL) corresponding to 10^6 ^cells.

### Cancer cell inoculation into nude mice

Eight-week-old male athymic nu/nu mice (Harlan, Germany) were used to produce orthotopic and subcutaneous prostate tumors. The animal experiments were carried out according to the European Convention for the Protection of Vertebrate Animals used for Experimental and other Scientific Purposes, Statutes 1076/85 §and 1360/90 of the Animal Protection Law in Finland and EU Directive 86/609. The experimental procedures were reviewed by the local Ethics Committee on Animal Experimentation at the University of Turku and approved by the local Provincial State Office of Western Finland.

*Experiment I *was performed to study the effects of VEGFR3 blockade on prostate tumor growth. For this, mice were randomized according to the weight into two groups (22 and 23 mice with VEGFR3-Ig and lacZ-Ig, respectively). 30 minutes before inoculation of tumor cells, an analgesic drug (Temgesic, 0.3 μg/g, Schering-Plough Europe, Belgium) was injected subcutaneously. The mice were anesthetized by means of isofluran inhalation (1.5 - 3%, air flow 200 mL/min, Univentor 400 anesthesia unit, Zejtun, Malta) and placed in a supine position under a sterile cover. An incision was made three mm above the pubic symphysis and the bladder and seminal vesicles were carefully lifted to expose the dorsal prostate. Subsequently, 5 × 10^5 ^PC-3 cells with green dye were slowly inoculated into the prostate. If leakage of cell suspension with green dye was observed in the peritoneal cavity or urethra/bladder, the mouse was not included in the experiment [32,33].

*Experiment II *was performed in order to study the effects of ectopic overexpression of VEGFR3 ligand, VEGF-C, on prostate tumor growth. First, the tumorigenicity of PC-3/VEGF-C cells was studied by means of subcutaneous inoculation. For this, mice were randomized according to weight into two groups (8 and 12 mice with PC-3/VEGF-C and PC-3/mock, respectively). Tumor cell suspension was inoculated subcutaneously at the back of the neck. After inoculation, tumors were measured twice a week and the volume was calculated according to formula (π/6)(d_1 _× d_2_)^3/2^, where d_1 _and d_2 _are perpendicular tumor diameters. To further study the effects of VEGF-C overexpression, the PC-3/VEGF-C cells were orthotopically inoculated into the prostates of nude mice as described above. For this, mice were randomized according to weight into two groups (*n = *33 and 28, PC-3/VEGF-C and PC-3/mock, respectively).

Next, we studied if we can block the VEGFR3 pathway in VEGF-C overexpressing orthotopic tumors. Mice were divided into two groups according to the weight and were injected with fusion proteins (12 and 8 mice with VEGFR3-Ig and lacZ-Ig, respectively) five minutes before orthotopic inoculation of PC-3/VEGF-C cells.

### In vivo delivery of VEGFR3-Ig by adenoviral vectors

Recombinant adenoviruses (1.2 × 10^8 ^pfu per mouse [35], a kind gift from Professor Seppo Ylä-Herttuala) [36] expressing the VEGFR3-Ig fusion protein (VEGFR-3Ig) or β-galactosidase (lacZ) as a control were administered via tail vein 5 minutes before orthotopic PC-3 or PC-3/VEGF-C cell inoculation.

### Histology and histomorphometry

Mice were sacrificed 4 weeks after subcutaneous or orthotopic inoculation of tumor cells. Subcutaneous tumor diameters were measured and tumors were removed. The prostates of orthotopic prostate tumor-bearing mice were exposed and prostate/tumor volume was calculated by means of the formula (d_1 _× d_2 _× d_3_)π/6 [37]. The prostate, the lungs and the regional prostate-draining lymph nodes (iliac and sacral) were macroscopically examined for the occurrence of tumors and subsequently collected for histology. Immediately after removal, tissue samples were immersed in 4% neutral-buffered formalin. After two to three days, the samples were transferred to 70% EtOH and stored until processed further. All samples were embedded in paraffin, cut through to five μm thick sections and stained with hematoxylin and eosin (H&E) using standard techniques. All samples were blind-evaluated for metastasis by two independent analyzers.

The iliac and sacral lymph nodes were cut through and 8 to 10 sections/lymph node at 200 μm intervals from every tumor-bearing mice were analyzed. The relative area of tumor metastasis in lymph nodes was determined from H&E-stained sections by means of an analysis software (AxioVision 4.3, Carl Zeiss Microimaging GmbH, Oberkochen, Germany). The results were expressed as percentage of the total area of the lymph node.

### Immunohistochemistry

Formalin-fixed, paraffin-embedded sections of prostate and lymph nodes were used for immunohistochemical studies. Five-μm sections were deparaffinized in xylene and rehydrated in a descending series of ethanol (100 - 70%). Endogenous peroxidase activity was blocked with 3% hydrogen peroxidase for 30 min at room temperature. Sections were incubated in humid chambers with a mouse LYVE-1 (a kind gift from Dr. David Jackson [38]), CD34 (Santa Cruz, USA), VEGFR3 (a kind gift from Professor Kari Alitalo [39]) and VEGFR2 (R&D, UK) antibodies o/n at 4°C. The samples were treated with biotin-labeled goat anti-mouse (Vector, Burlingame, USA), a biotin-labeled rabbit anti-rat (DAKO, Denmark) or biotin-labeled rabbit anti-mouse secondary antibodies, according to the nature of the primary antibodies. Immunoperoxidase staining was performed using an ABC kit (Vector Laboratories, CA, USA). The slides were stained with diaminobenzidine (DAB) and counterstained with Meyer's hematoxyline.

Known positive samples from every tumor and negative controls (sections of every sample stained without the primary antibody) were used to verify the specificity of staining. Vascularization was determined as the density of the CD34-, m-LYVE-1-, VEGFR2- and VEGFR3-positive vessels. Vessels were determined from the three representive, non-overlapping fields from one section of each sample by drawing lines following stained vessels [32] and measuring the density using AxioVision 4.3 software (Carl Zeiss Microimaging GmbH, Oberkochen, Germany). The results were blind-tested by two independent analyzers.

### Statistics

Results were expressed as mean value ± SEM. Statistical significance was taken as *p *< 0.05 using two-tailed Student's *t*-tests and chi square tests.

## Results

### Effect of VEGFR3-Ig fusion protein on tumor vessels and lymph node metastasis

**Figure 1:**
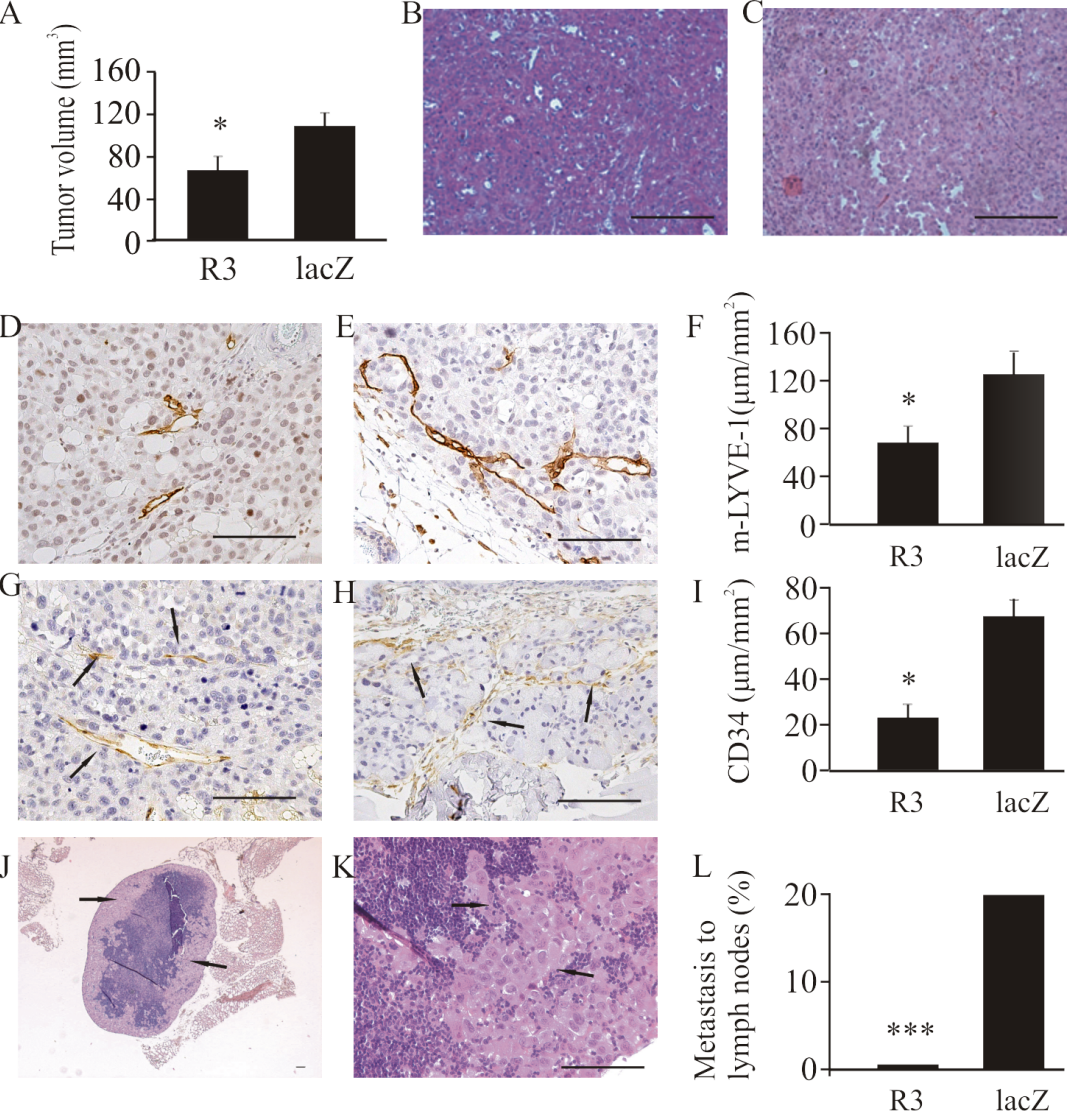
**Effects of adenoviral VEGFR3-Ig fusion protein on growth and metastasis of orthotopic PC-3 tumors**. Mice were treated with recombinant adenoviruses expressing the VEGFR3-Ig fusion protein (R3) or β-galactosidase (lacZ). Tumor occurrence was 68% (*n *= 15/22) in the VEGFR3-Ig group and 91% (*n *= 21/23) in the control (lacZ-Ig) group. Blocking of VEGFR3 decreased tumor volume compared with those in control vector treated mice (A, VEGFR3-Ig 77 ± 6 mm^3^, *n *= 15 *vs*. lacZ-Ig 120 ± 8 mm^3^, *n *= 21, *p *< 0.05). No major differences in morphology were detected in H&E-stained sections of tumors from the VEGFR3-Ig (B) *vs*. lacZ-Ig treated mice (C). Histomorphometric analysis of tumor sections immunostained with m-LYVE-1 antibody demonstrated that blocking of VEGFR3 decreased the density of lymphatic capillaries (D, VEGFR3-Ig 69 ± 19 μm/mm^2^, *n *= 15 *vs*. E and F, lacZ-Ig 130 ± 18 μm/mm^2^, *n *= 21, *p *< 0.05). Immunohistochemical staining of tumor sections with CD34 antibody showed that blocking of VEGFR3 decreased the density of blood capillaries, arrows (G, VEGFR3-Ig 24 ± 5 μm/mm^2^, *n *= 15 *vs*. H and I, lacZ-Ig 67 ± 8 μm/mm^2^, *n *= 21, *p *< 0.05). The incidence of metastasis (arrows) in prostate-draining lymph nodes was 0% (*n *= 0 out of 15 tumor bearing mice) in the VEGFR3-Ig group and 19% (*n *= 4 of 21 tumor bearing mice, *p *< 0.001) in the control group (J-L). The occurrence of metastasis in lungs was 0% in both groups (data not shown). Bar 500 μm.

Human prostate cancer cells of the PC-3 line endogenously express VEGF-C [9]. PC-3 cells were inoculated into the prostates of nude mice to produce orthotopic tumors. We and others have previously shown that they metastasize to iliac and sacral lymph nodes [32,40]. In order to block VEGF-C signaling, mice were injected with recombinant adenoviruses expressing soluble VEGFR3-Ig fusion protein (VEGFR3-Ig) that binds VEGF-C [36]. A β-galactosidase fusion protein (lacZ-Ig) was used as a control. Mice were sacrificed 4 weeks after inoculation and the effects of VEGFR3-Ig on tumor growth, morphology, lymphangiogenesis, angiogenesis and metastasis to the prostate-draining iliac and sacral lymph nodes and the lungs were studied. Tumor occurrence in the prostate was 15/22 (68%) and 21/23 (91%) in the VEGFR3-Ig and lacZ-Ig groups, respectively. The mean size of PC-3 tumors was significantly smaller in mice injected with VEGFR3-Ig than in lacZ-Ig-injected control mice (77 ± 6 mm^3 ^*vs*. 120 ± 8 mm^3^, respectively, *p *< 0.05, Fig 1A).

Histological staining (H&E) did not reveal major differences in tumor morphology (Fig 1B-C). Lymphatic vessels were visualized using immunohistochemical staining with m-LYVE-1 antibody and blood vessels using CD34 antibody. A moderate network of capillaries was seen in all tumors, but treatment with VEGFR3-Ig significantly decreased the density of both lymphatic (Fig 1D-F, 69 ± 19 μm/mm^2 ^*vs*. 130 ± 18 μm/mm^2^, *p *< 0.05) and blood vessels in tumors (Fig 1G-I, 24 ± 5 μm/mm^2 ^*vs*. 67 ± 8 μm/mm^2^, *p *< 0.05).

Histological analysis of prostate-draining lymph nodes showed that VEGFR3-Ig treatment effectively inhibited lymph node metastasis. The incidence of metastases at 4 weeks was 19% in the lacZ-Ig-injected tumor bearing mice but null in the VEGFR3-Ig mice (Fig 1J-L, *p *< 0.001). No metastases were found in the lungs (data not shown).

### Ectopic expression of VEGF-C in PC-3 cells

**Figure 2:**
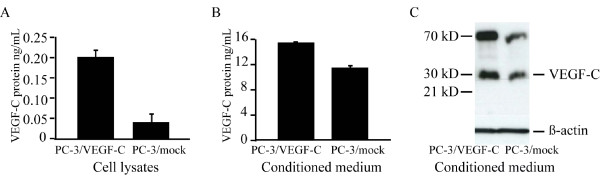
**Ectopic expression of VEGF-C in PC-3 cells and orthotopic tumors**. PC-3 cells were stably transfected with a vector containing human VEGF-C (PC-3/VEGF-C) or empty vector (mock). One cell clone expressing VEGF-C at a high level, and one control clone (PC-3/mock) were selected for further studies. ELISA analysis of VEGF-C protein concentration in cell lysates (A) and in conditioned media (B) revealed increased expression in PC-3/VEGF-C cells compared with PC-3/mock cells. Data are expressed as means ± SD ng/mL, corresponding 10^6 ^cells. (C) Western blot analysis revealed that PC-3 cell clones expressed mainly 29/31 kD form of VEGF-C. Detectable levels of 21 kD form were not seen. β-actin control was from cell lysates corresponding to the collected conditioned medium.

In order to study the effect of VEGF-C overexpression on tumor growth and metastasis, PC-3 cells were transfected with a pcDNA3.1(+) vector containing the full coding sequence for VEGF-C [9]. Several positive clones were obtained. One clone expressing VEGF-C mRNA (data not shown) and protein at a high level were selected for tumor experiments (Fig 2, PC-3/VEGF-C). ELISA analysis of the cell lysates showed difference between PC-3/VEGF-C and PC-3/mock (0,2 ng/mL ± 0.03 in PC-3/VEGF-C cells and 0.04 ng/mL ± 0.02 in PC-3/mock cells, corresponding to 10^6 ^cells, Fig 2A). VEGF-C is a rapidly secreted protein, and the analysis of media conditionedfor 24 h confirmed that PC-3/VEGF-C cells secrete increased levels of VEGF-C protein compared with PC-3/mock (14.4 ng/mL ± 0.1 and 10.6 ng/mL ± 0.2, respectively, Fig 2B). The VEGF-C ELISA detects all human VEGF-C forms including full-length VEGF-C proteolytically processed forms with different biological activities and affinities for VEGF receptors. Western blot analysis of the conditioned medium showed that the 29/31 kD VEGF-C polypeptides were the major partially processed forms (Fig 2C). Small 21 kD form was not detected.

### Effect of VEGF-C on growth and metastasis of orthotopic and subcutaneous PC-3 tumors

**Figure 3:**
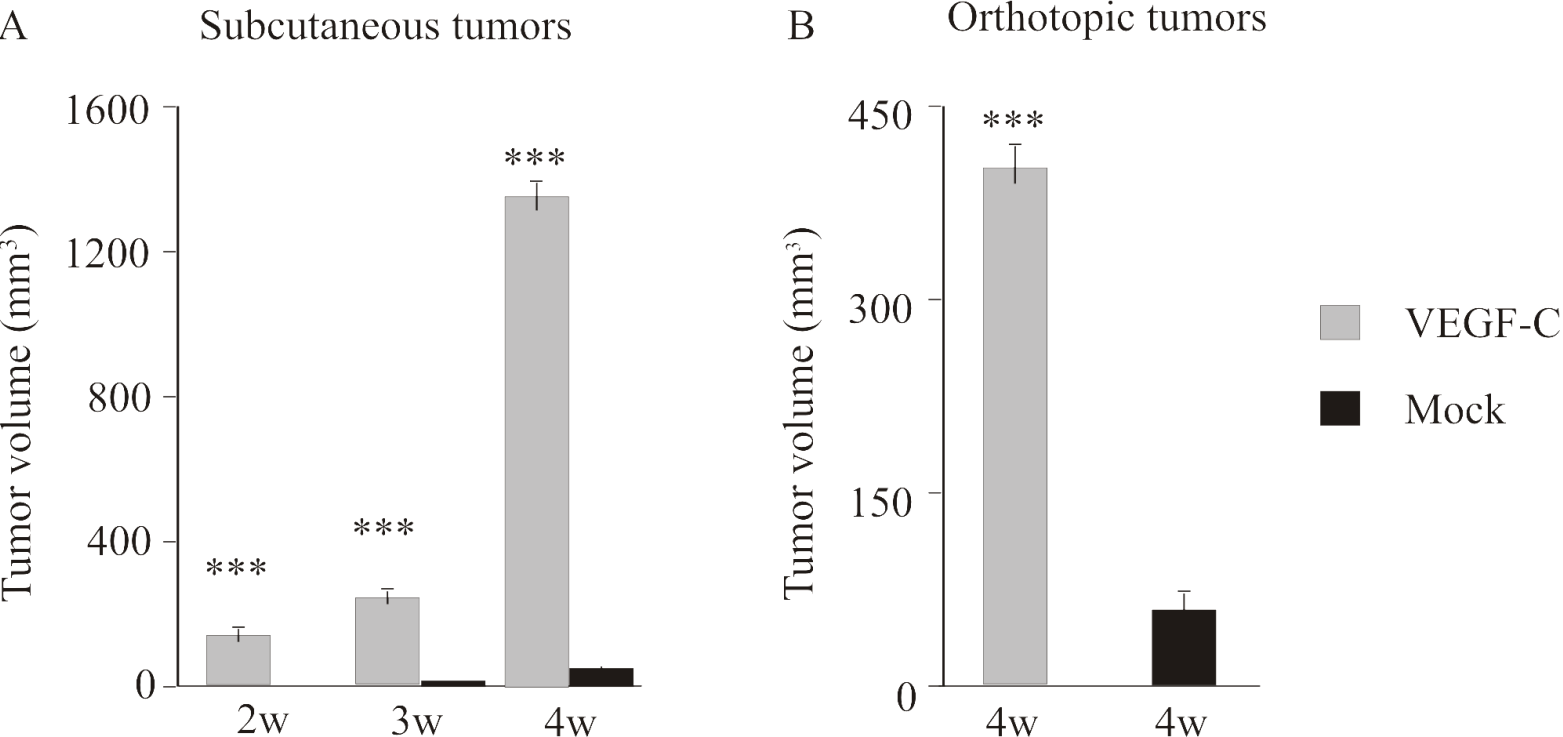
**Effect of VEGF-C overexpression on PC-3 prostate tumor growth**. The occurrence of subcutaneous tumors of both PC-3/VEGF-C (*n *= 8) and PC-3/mock (*n *= 12) was 100%. (A) Subcutaneous PC-3/VEGF-C tumors grew larger (1132 ± 34 mm^3^, gray bars) than PC-3/mock tumors (47 ± 7 mm^3^, black bars) at 4 weeks and the differences in tumor volumes between the groups were significant at all time points between 2 and 4 weeks (*p *< 0.001). (B) The occurrence of orthotopical tumors was 88% (*n *= 29 out of 33 mice) in the PC-3/VEGF-C group and 86% (*n *= 24 out of 28 mice) in the control group. The mean volume of the PC-3/VEGF-C tumors was 400 ± 17 mm^3 ^(*n *= 29) and in PC-3/mock tumors it was 48 ± 4 mm^3 ^(*n *= 24), *p *< 0.001.

To investigate tumor growth, PC-3/VEGF-C and PC-3/mock cells were inoculated subcutaneously into the neck or orthotopically into the prostate of nude mice. The experiment was ended at 4 weeks. Ectopic expression of VEGF-C increased subcutaneous tumor growth (*p *< 0.001 at all time points between 2 to 4 weeks, Fig 3A). At 4 weeks, the PC-3/mock tumors were small (47 ± 7 mm^3^) while the PC-3/VEGF-C tumors were 24 × larger (1132 ± 34 mm^3^).

The occurrence of orthotopic prostate tumors was 29/33 (88%) in the PC-3/VEGF-C group and 24/28 (86%) in the PC-3/mock group, when they were measured in the end of the experiment. As in the subcutaneous tumors, the orthotopic tumors overexpressing VEGF-C were markedly bigger (400 ± 17 mm^3 ^*vs*. 48 ± 4 mm^3^, *p *< 0.001, Fig 3B).

### Effect of VEGF-C on lymphatic capillaries

**Figure 4:**
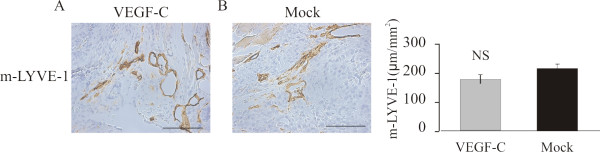
**Effect of ectopic VEGF-C on lymphatic vessel density of orthotopic PC-3 tumors**. M-LYVE-1-positive staining was observed primarily in the tumor periphery and in the peritumoral area (A-B). No differences were detected in the density of lymphatic vessels Bar 500 μm.

The analysis of the distribution of lymphatic vessels showed that lymphatic vessels stained for m-LYVE-1 were located mainly in tumor periphery (Fig 4A). When the densities of lymphatic vessels were calculated and the peripheral areas of the tumors were compared, there was no statistically significant difference between PC-3/VEGF-C (Fig 4A) and PC-3/mock (Fig 4B) tumors. The distribution of lymphatic vessels was also studied using an antibody against VEGFR3. Again positive staining was seen mainly in the peripheral areas and tumor periphery. No statistically significant difference in distribution of VEGFR3 positive vessels was detected between PC-3/VEGF-C *vs*. PC-3/mock tumors or PC-3(VEGFR3-Ig) *vs*. PC-3(lacZ-Ig) tumors (data not shown).

### Increased angiogenesis in orthotopic PC-3 tumors expressing ectopic VEGF-C

**Figure 5:**
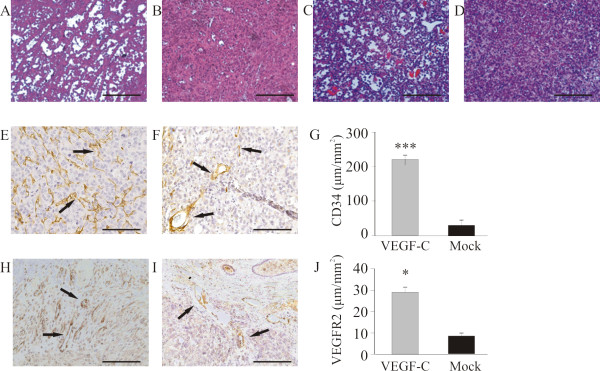
**Effect of VEGF-C overexpression on angiogenesis of orthotopic tumors**. H&E-staining of representative PC-3/VEGF-C (A, orthotopic; C, subcutaneous) tumors and PC-3/mock (B, orthotopic; D, subcutaneous) tumors. PC-3/VEGF-C tumors showed angiogenic morphology with a rich network of capillaries compared with PC-3/mock tumors. There were significantly more blood capillaries (CD34 positive, arrows) in the PC-3/VEGF-C tumors (E, 220 ± 15 μm/mm^2^, *n *= 29) compared with PC-3/mock tumors (F and G, 37 ± 6 μm/mm^2^, *n *= 24), *p *< 0.001. Density of VEGFR2-positive capillaries was analyzed similarly. Again there were significantly more VEGFR2-positive capillaries in the PC-3/VEGF-C tumors (H, 30 ± 0.3 μm/mm^2^, *n *= 29) than in the PC-3/mock tumors (I and J, 9 ± 0.1 μm/mm^2^, *n *= 24), *p *< 0.05. Bar 500 μm.

The morphology of VEGF-C-expressing subcutaneous and orthotopic tumors differed greatly from that of PC-3/mock tumors. H&E staining of the PC-3/VEGF-C tumors showed a rich network of capillary-like structures (Fig 5A and 5C), whereas PC-3/mock tumors were homogeneous and solid with moderate vascular network (Fig 5B and 5D). The density of blood capillaries was studied by means of immunohistochemical staining for CD34 (Fig 5E and 5F). Fig 5G shows that the density was indeed significantly higher in VEGF-C overexpressing tumors compared with PC-3/mock tumors (220 ± 15 μm/mm^2 ^*vs*. 37 ± 6 μm/mm^2^, *p *< 0.001). The density of VEGFR2-positive vessels was also higher in the PC-3/VEGF-C tumors compared with PC-3/mock tumors (*p *< 0.05) (Fig 5H, I and 5J, 30 ± 0.2 μm/mm^2 ^*vs*. 9 ± 0.1 μm/mm^2^). Capillary densities were also calculated in the metastatic areas of prostate-draining lymph nodes of PC-3/VEGF-C and PC-3/mock tumors but no difference between the groups was observed (data not shown).

### Metastasis of VEGF-C-overexpressing tumors

**Figure 6:**
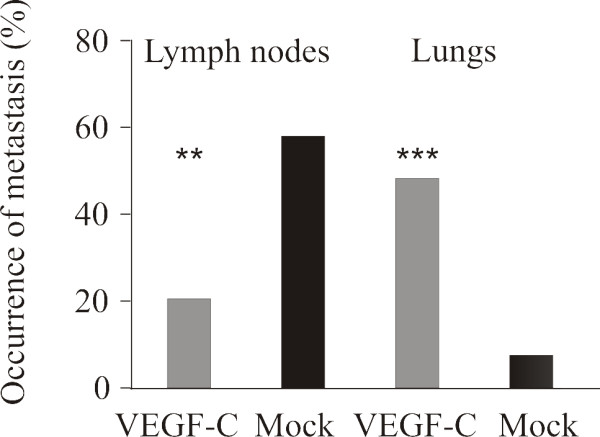
**Metastasis of PC-3/VEGF-C tumors to prostate-draining lymph nodes and lungs**. The prostate draining lymph nodes and the lungs were cut through to detect metastasis. Altogether 8-10 lymph nodes and six lung sections/mouse were studied and number of metastases was counted. The relative metastatic area of lymph nodes was determined by histomorphometry but there were no statistically significant differences (data not shown). The occurrence of metastasis in lymph nodes was 21% (*n *= 6 mice out of 29 tumor-bearing mice) in the PC-3/VEGF-C group and 58% (*n *= 14 mice out of 24 tumor-bearing mice) in the PC-3/mock group and in lungs was 48% (*n *= 14 out of 29 tumor bearing mice) in the PC-3/VEGF-C group and 8% (*n *= 2 out of 24 tumor-bearing mice) in the PC-3/mock group.

The incidence of lymph node metastases was studied four weeks after orthotopic inoculation of PC-3/VEGF-C and PC-3/mock cells by histologic evaluation of H&E staining and histomorphometry of the serially sectioned lymph nodes. Surprisingly, metastases in iliac and sacral lymph nodes were found only in 21% of the mice bearing PC-3/VEGF-C tumors compared with 58% of those with PC-3/mock tumors (*p *< 0.01, Fig 6). Histomorphometric analysis of lymph nodes did not show significant differences in the relative metastatic tumor areas in iliac and sacral lymph nodes between the groups 4 weeks after inoculation of tumor cells (data not shown). Interestingly, however, histologic evaluation of the lungs showed that the incidence of metastasis was markedly increased in the VEGF-C-overexpressing group: 48% of mice inoculated with PC-3/VFGF-C cells had metastases in the lungs compared with 8% of the control mice (*p *< 0.001, Fig 6).

### VEGFR3-Ig fusion protein treatment decreased the density of lymphatic capillaries

**Figure 7:**
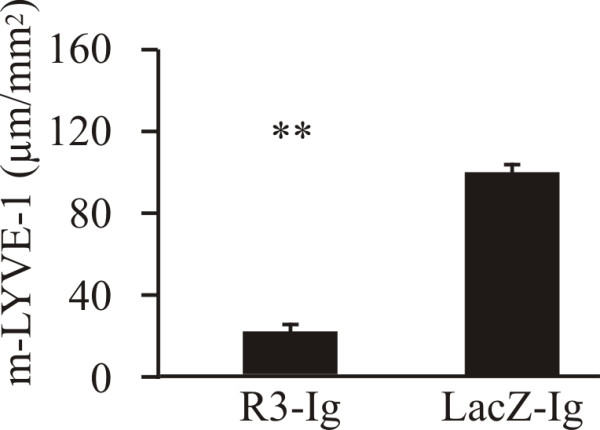
**Effect of VEGFR3-Ig treatment on orthotopic PC-3/VEGF-C tumors**. VEGFR3-Ig fusion protein-producing construct or lacZ-Ig controls were injected intravenously into mice five minutes before orthotopic inoculation of PC-3/VEGF-C cells. The density of m-LYVE-1 positive lymphatic capillaries was decreased in VEGFR3-Ig-treated tumors (23 ± 4 μm/mm^2^, *n *= 8 *vs*. 123 ± 7 μm/mm^2^, *n *= 6, *p *< 0.05).

A group of mice inoculated with PC-3/VEGF-C cells was also treated with VEGFR3-Ig fusion protein or lacZ-Ig to study whether blockade of the VEGFR3 pathway is able to oppose the effects of VEGF-C overexpression. As in parental PC-3 tumors treated with VEGFR3-Ig (Fig 1), the density of lymphatic capillaries was decreased in VEGFR3-Ig-treated prostate tumors compared with lacZ-Ig-treated (Fig 7, 23 ± 4 μm/mm^2^*vs*. 123 ± 7 μm/mm^2^, *p *< 0.01). There was also a trend for a decreased number of blood capillaries as well as decreased size in VEGFR3-Ig-treated prostate tumors but the difference did not reach a statistical significance (data not shown).

## Discussion

Expression of VEGF-C and VEGFR3 has been shown to correlate with clinical markers such as concentration of the circulating prostate-specific antigen (PSA) and the Gleason score in prostate cancer [13,15]. However, it is not clear whether VEGF-C is only a marker of progressed disease or if it also facilitates lymph node metastasis in prostate cancer. Experimental models suggest that VEGF-C is involved in tumor lymphangiogenesis and often, but not always, it is upregulated in metastatic tumors [10-12,41-43]. According to some reports, VEGF-C-stimulated lymphangiogenesis in tumors and lymph nodes is required for metastasis at distant sites [44]. On the other hand, Padera *et al*. [22] and Wong *et al*. [23] have shown that lymph node metastasis may develop without increased lymphangiogenesis and or even absence of intratumoral lymphatic vessels [23].

We used orthotopic PC-3 prostate tumors that metastasize to regional lymph nodes [32,40] to study the role of VEGF-C in prostate tumor growth and metastasis. Blockade of VEGFR3 with VEGFR3-Ig decoy receptor protein led to a decreased density of lymphatic capillaries, which was associated with total inhibition of lymph node metastasis. These results are in line with those of previous studies on prostate cancer [13-15,31] and other prostate tumor models [45] suggesting that VEGF-C signaling via VEGFR3 has a major role in lymph node metastasis. Some of the previous studies also suggest correlation between the density of lymphatic vessel network and spreading of tumor cells to lymph nodes, although other investigators have not found an association between lymphatic vessel density and lymph node metastasis [22,23,46].

In our study, the blockade of VEGFR3 by VEGFR3-Ig also inhibited the growth of orthotopic PC-3 tumors and decreased the density of blood vessels as shown by quantification of CD34-immunostained capillaries within tumors. The VEGFR3 has been shown to contribute to the formation of blood capillaries in various tumor models [47], which may explain the effects on angiogenesis as well as the decreased tumor size in orthotopic PC-3 tumors. Accordingly, orthotopic PC-3 tumors overexpressing ectopic VEGF-C grew much faster than mock-transfected controls. The morphology of the tumors was highly angiogenic as shown by histology and immunohistochemical staining for CD34 and VEGFR2. This could be explained by the fact that VEGFR3 also contributes to angiogenesis as suggested in another study on PC-3 tumors [47]. It has been shown that signaling via VEGFR3 stimulates angiogenesis in developing tissues and at least in some tumors [48]. This possibility is also supported by our observation of decreased angiogenesis in tumors in which VEGFR3 was blocked with VEGFR3-Ig decoy receptor. Other possible explanations could be the ability of VEGF-C to bind and activate VEGFR2, which is primarily expressed in blood capillary endothelium [6], or stimulation of angiogenic VEGFR2 by heterodimerisation of VEGFR3 with VEGFR2 [6,49]. PC-3 cells transfected with the VEGF-C gene almost exclusively expressed and secreted a proteolytically processed form of VEGF-C (29/31 kDa) that primarily binds VEGFR3 but also VEGFR2 even if with minor affinity [6].

Analysis of the distribution of the lymphatic capillaries showed that there was no statistically significant difference in the density of lymphatic capillaries in tumor periphery between PC-3/VEGF-C and PC-3/mock tumors. However, it seemed that the center of PC-3/VEGF-C tumors had fewer lymphatic vessels than PC-3/mock and PC-3 tumors. Other investigators have reported that tumor center lacks functional lymphatic vessels [22,23] or that it is primarily the peripheral lymphatic capillaries that mediate tumor cell spread to lymph nodes [23]. Many studies have also reported an increased network of peritumoral lymphatic vessels in experimental breast and prostate tumors [10-20]. It has been claimed that elevated interstitial fluid pressure within the tumors may compress thin-walled lymphatic capillaries and compromise their function [50]. In our experiments it seemed likely that the highly expanded network of blood vessels in PC-3/VEGF-C tumors may have caused pressure that has impaired or prevented growth of lymphatic capillaries within the tumor tissue. This may also have caused decreased metastasis to lymph nodes. However, our observation that the presence of peritumoral lymph vessel network similar to that in control tumors was associated with decreased lymph node metastasis. This suggests that also the central vessels play a role in lymphatic tumor spread or that other metastatic routes were primarily used in PC-3/VEGF-C tumors.

Interestingly, while high expression of VEGF-C in orthotopic PC-3 tumors was associated with decreased lymph node metastasis, the rate of lung metastasis was clearly increased. Even wild-type PC-3 tumors metastasize to the lungs, but at a low rate and slowly along an extended time period whereas in PC-3/VEGF-C tumor-bearing mice metastasis to the lungs was increased or at least clearly accelerated. In our experiments it seems possible that in these tumors, lung metastases arose via hematogenous pathways, facilitated by the extensive blood capillary network. Another possibility is that the tumor cells spreading via lymphatic vessels passed the lymph nodes and were taken to distant sites. Burton *et al*. [21] very recently reported that in different orthotopic prostate cancer models VEGF-C/VEGFR3 mediated metastasis to lymph nodes was mandatory to metastasis to distant sites. In addition, Hirakawa *et al*. [44] have recently shown evidence that VEGF-C can facilitate tumor cell metastasis to lung and other organs by modulating lymph node vascularization and function. If this was the case in our PC-3/VEGF-C tumor-bearing mice, the reasonably dense network of peritumoral lymphatic vessels would have mediated the spread of the tumor cells to distant sites via lymph nodes to the lungs. It will be interesting to investigate in further studies the possible effect of VEGF-C on tumor cell survival outside the tumor environment and on the interactions of tumor cells with lymphoid tissues and cells at other metastatic sites.

Our observations on a switch of a lymphangiogenic to an angiogenic-type pattern of metastasis upon high VEGF-C expression in prostate cancer models differ from the results of some other studies [20,21]. Those studies reported that VEGF-C/VEGFR3 stimulation primarily increased lymphatic vessel network and lymph node metastasis, which was also a prerequisite for later lung metastasis if it was observed. The reason for this discrepancy is presently unclear but one explanation in our tumors could be a expanded blood capillary network, which may have provided a more effective pathway for metastasis than lymphatic capillaries. It is obvious, however, that the regulation of metastatic pathways by different factors is very versatile and context dependent in various tumor models [23,48].

Treatment of PC-3/VEGF-C tumor bearing mice with VEGFR3-Ig further decreased the density of lymph vessels. This suggests that the formation of new lymphatic capillaries in PC-3 tumors is dependent on VEGFR3 function even in the presence of massive angiogenesis. Treatment with VEGFR3-Ig had, however, less obvious effects on CD34-positive blood capillary density and tumor size than in parental PC-3 tumors. The reason for this difference is presently unclear but it is possible that the VEGFR3-Ig treatment conditions (concentrations, dosing schedule) should have been further scrutinized to obtain an optimal inhibition of all effects of VEGF-C overexpression.

## Conclusion

Our results show that although the VEGF-C/VEGFR3 pathway is important for formation of lymphatic vessels and lymph node metastasis of orthotopic PC-3 prostate tumors, high expression level of VEGF-C can also lead to activation of angiogenesis. This is associated with a change in the pattern of primary lymph node metastasis to preferential occurrence of metastasis at distant sites such as the lung, which suggests that the hematogenous route is preferred over the lymphatic route as a metastatic pathway of the orthotopic prostate tumors. Our results support the concept that the development of angiogenic and lymphangiogenic metastatic routes is tumor and context dependent. An effective blockade of both lymphangiogenic and hematogenous pathways is thus needed to inhibit metastasis in prostate cancer.

## Abbreviations

cDNA: complementary deoxyribonucleic acid; DAB: diaminobenzidine; DMEM: Dulbecco's modified Eagle's medium; EDTA: ethylenediaminetetra-acetic acid; H&E: hematoxylin-eosine; iFBS: heat-inactivated fetal bovine serum; iv: intravenous; lacZ: β-galactosidase fusion protein; mRNA: messenger ribonucleic acid; PSA: prostate-specific antigen; PBS: phosphate-buffered saline; s.c.: subcutaneous; VEGF: vascular endothelial growth factor; VEGF-C: vascular endothelial growth factor C; VEGF-D: vascular endothelial growth factor D; VEGFR2: vascular endothelial growth factor receptor 2; VEGFR3: vascular endothelial growth factor receptor 3; VEGFR3-Ig: vascular endothelial growth factor receptor 3 fusion protein.

## Competing interests

The authors declare that they have no competing interests.

## Authors' contributions

JT carried out the cell cultures, RNA isolation, Northern Blot analyses, analysis of morphology and morphometry and statistical analysis. Western Blot analyses and ELISA was performed by JT and KT. Transfection of the PC-3 cell was done by JS. Orthotopic tumor experiments were done by JT, MV and JS. PH and KV participated in the design of the study. JT wrote the first version of the manuscript and all authors helped to process it. All authors have read and approved the final manuscript. PH gave final approval for the manuscript to be submitted.

## Pre-publication history

The pre-publication history for this paper can be accessed here:

http://www.biomedcentral.com/1471-2407/9/362/prepub
